# P323: Epidemiological study of drug administration routes (dar) in the department of pediatrics, Gabriel Touré hospital. Mali

**DOI:** 10.1186/2047-2994-2-S1-P323

**Published:** 2013-06-20

**Authors:** M Daouda, AS Kaya, B Traoré, L Bengaly, M Sacko, A Camara, AM Traoré, M Diakité, H Cissé, AT Sidibé, HA Traoré, T Sidibé, MM Keita

**Affiliations:** 1 Service of Infectious Diseases,Department of Public Health, Faculty of Medicine, Bamako, Mali; 2 Department of Internal medecine, University Hospital of Point G, Department of Public Health, Faculty of Medicine, Bamako, Mali; 3 Pediatric Services, Department of Public Health, Faculty of Medicine, Bamako, Mali; 4 Hospital Pharmacy, CHU Gabriel Touré, Department of Public Health, Faculty of Medicine, Bamako, Mali; 5 Department of Public Health, Faculty of Medicine, Bamako, Mali

## Objectives

Injectable route seems to be the most frequently used in health facilities in a resource-limited setting.

## Methods

In order to better understand this under investigated issue, we conducted a longitudinal study of DAR use in the general pediatric ward of the Gabriel Touré Hospital to Bamako during 6 months.

## Results

We are interested in the routes of administration applied to a population of 300 children with a sex ratio (M / F) = 1.3. Their average age was 2 years ± 1. Presumed diagnoses underwent a change from admission to discharge, both in frequency and formulation. Malaria (37.4% vs 39.7% at 72 hours), pneumonia (19% vs 20% at 72 hours), and the nephrotic syndrome (2.2% vs. 5.1% in 72 hours) were most commonly mentioned. Treatments prescribed for the presumed diagnosis were administered parenterally in 76.6% of cases at admission, in 70% 72h hours after admission and in 36.3% at discharge. The following complications were noted: inflammation of the catheter puncture sites (21.8% at admission, 18% after 5 days of hospitalization), abscess at the site of intramuscular injection (2.1%). The mean duration of hospitalization was 7.6 ± 3.7 days and mortality was 11%.

## Conclusion

Injection is the most widely used in the pediatric unit III for the most common pathologies. A detailed study would be needed to assess the adequacy of the diagnostic hypotheses and routes of administration. The high number of injections exposes staff and patients to risks.

## Disclosure of interest

None declared

